# Endoscopic ultrasound-guided and submucosal tunnel-assisted endoscopic removal of a long foreign body incarcerated in the duodenal bulb and abdominal cavity

**DOI:** 10.1055/a-2825-8559

**Published:** 2026-03-24

**Authors:** Qi Gong, Hua Huang, Yiying Qi, Jinling Cheng, Min Li, Fuyan Wang, Zhi Wei

**Affiliations:** 1 611300 Department of Gastroenterology, Shandong Second Provincial General Hospital, Jinan, China

**Fig. 1:**
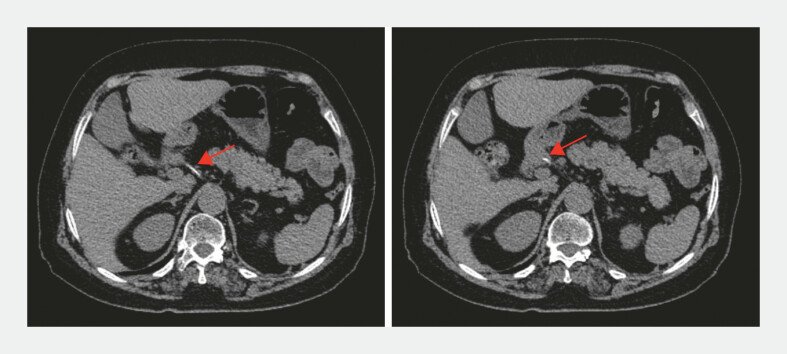
Computed tomographic scan showing a high-density shadow foreign body incarcerated in the duodenal bulb and abdominal cavity (red arrow).

**Fig. 2:**
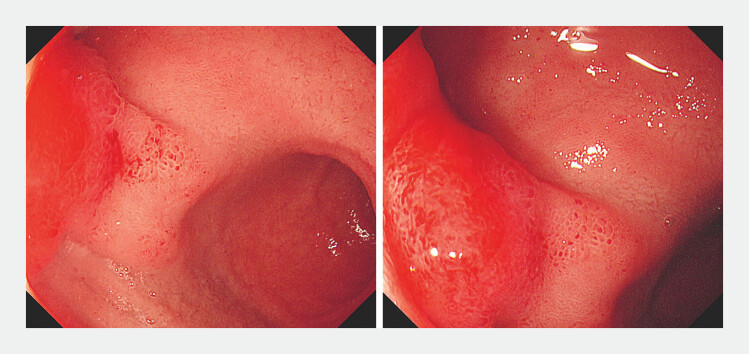
Gastroscopy revealed that a bulge was observed in the duodenal bulb with visible congestion and edema on top.

**Fig. 3:**
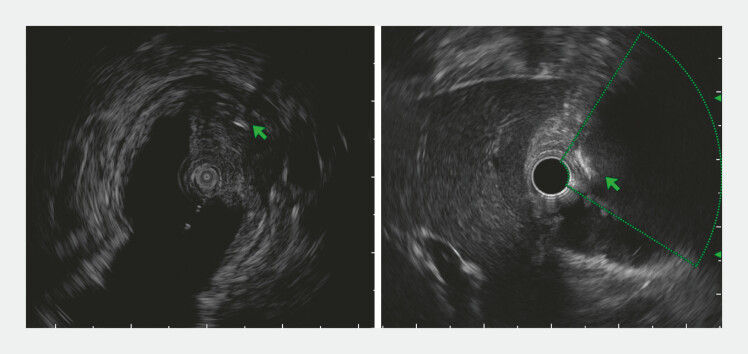
EUS was used to locate the striped hyperechoic linear foreign body (green arrow). EUS, endoscopic ultrasound.

**Fig. 4:**
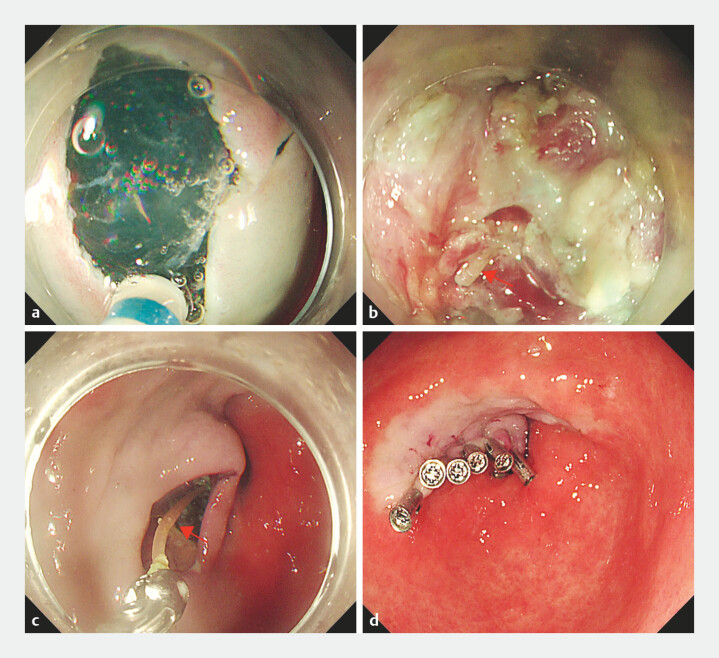
**a** A tunnel entrance was established on the anterior wall of the pylorus. **b** Fishbone embedded in the muscularis propria was revealed (red arrow). **c** The foreign body was retrieved using biopsy forceps (red arrow). **d** The tunnel entrance was closed with clips.

**Fig. 5:**
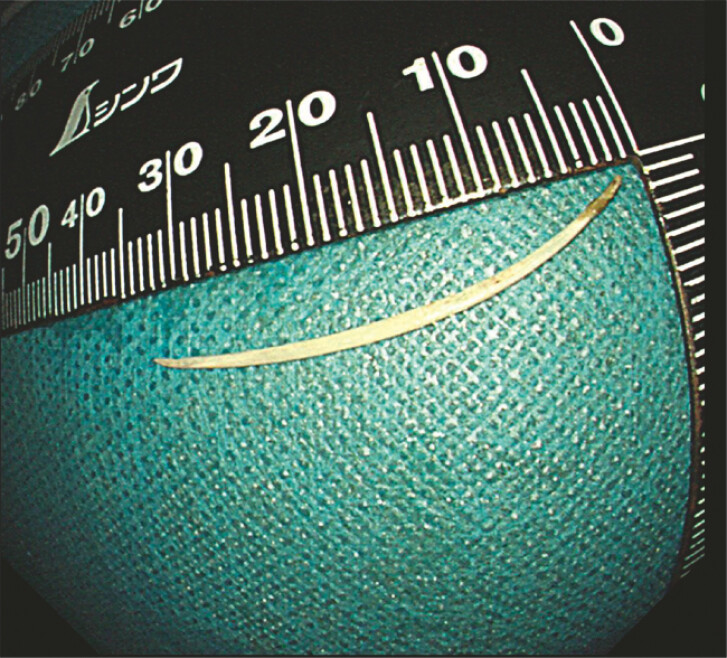
Approximately 3.3 cm fish bone.

A 77-year-old women presented to our hospital with a 2-week history of left-sided abdominal pain. Computed tomographic (CT) scanning showed a long foreign body (approximately 3.0 cm) incarcerated in the duodenal bulb and abdominal cavity ( Fig. 1 ). Gastroscopy revealed that a bulge was observed in the duodenal bulb with visible congestion and edema on top ( Fig. 2 ). The patient had accidentally swallowed a fishbone about a month ago and did not develop typical symptoms of acute duodenum penetration. Endoscopic ultrasound was used to locate the striped hyperechoic linear foreign body ( Fig. 3 ). Given the high surgical risk and uncertain prognosis, submucosal tunnel-assisted endoscopy was performed to remove the foreign body, and a mucosal incision knife was used to create the tunnel entrance on the anterior wall of the pylorus. The circular muscle of the pylorus was shallowly cut to the mucosal protrusion of the duodenal bulb, along the longitudinal axis of the linear foreign body ( Fig. 4 and Video 1 ). The fishbone was finally revealed and removed ( Fig. 5 ). The patient fasted for 1 day and was discharged after 3 days without any postoperative complications. A follow-up CT scan confirmed the absence of any residual foreign body.

Endoscopic ultrasound-guided and submucosal tunnel-assisted endoscopic removal of a long foreign body incarcerated in the duodenal bulb and abdominal cavity.Video 1

The extraction of sharp foreign bodies poses a persistent challenge in endoscopic intervention 1 . The European Society of Gastrointestinal Endoscopy (ESGE) recommends urgent endoscopic intervention for sharp-pointed foreign bodies, advising their prompt removal 2 . Due to the limited space in the duodenal bulb and the poor stability of the endoscope, direct endoscopic myotomy may increase the risk of perforation, which would require additional surgical intervention. We report the successful application of the endoscopic ultrasound-guided and submucosal tunnel-assisted endoscopic removal of a long foreign body incarcerated in the duodenal bulb and abdominal cavity. This integrated approach ensured that the integrity of the mucosa and sufficient operating space was maintained, provided a minimally invasive alternative, avoiding the need for surgery.

Endoscopy_UCTN_Code_TTT_1AO_2AG_3AD Endoscopy_UCTN_Code_CCL_1AB_2AZ_3AZ
